# Factors Associated With Digital Intervention Engagement and Adherence in Patients With Cancer: Systematic Review

**DOI:** 10.2196/52542

**Published:** 2024-12-11

**Authors:** Lucile Montalescot, Louise Baussard, Elodie Charbonnier

**Affiliations:** 1 APSY-V Université de Nîmes Nîmes France; 2 Laboratoire de Psychopathologie et Processus de Santé Université Paris-Cité Boulogne-Billancourt France

**Keywords:** adherence, engagement, eHealth, mHealth, cancer, mobile health, app, eHealth interventions, patient, cancer care, digital health, health-related, intervention-related, sociodemographic, behavior, systematic review

## Abstract

**Background:**

Digital interventions offer vital support for patients with cancer through education, behavior change, and monitoring. Despite their potential, patient adherence to and engagement with these self-help interventions is challenging. Factors like user characteristics, technology, and intervention design influence adherence and engagement. Existing reviews have gaps in exploring diverse factors associated with adherence in cancer care.

**Objective:**

This systematic review aims to identify factors influencing adherence to and engagement with digital interventions with self-help components in cancer care. It examined sociodemographic, psychosocial, health-related, and intervention-related factors that affect patients’ adherence to and engagement with these digital health solutions.

**Methods:**

Following PRISMA (Preferred Reporting Items for Systematic Reviews and Meta-Analyses) guidelines, a search was conducted across PubMed, Embase, Cochrane Library, and PsycINFO to find studies published from January 2010 to September 2021. The studies included in this review focused on adult patients with cancer using digital interventions with self-help features. Data were extracted and synthesized using a standardized approach. Factors associated with adherence were synthesized according to their type—sociodemographic factors, psychosocial factors, health-related factors, technology-related factors, and intervention-related factors.

**Results:**

Among 9386 studies initially screened, 61 (0.6%) were eligible for analysis. These studies covered diverse eHealth intervention types, cancer types, and outcome measures. Investigating the determinants of adherence to and engagement with digital interventions was the main objective for 43% (26/61) of the included studies. Adherence and engagement were gauged using varied measures, such as dropout rates, log-ins, and self-reported measures. Results regarding factors associated with adherence and engagement were inconsistent across studies. Most sociodemographic (eg, age) and health-related factors (eg, cancer stage) yielded mixed outcomes. However, comorbidity consistently predicted lower adherence and engagement. Results regarding psychosocial factors were more stable across studies. Specifically, higher social support was associated with lower adherence and engagement. Finally, intervention-related factors like intervention type or human support showed conflicting results. Adopting an intersectional perspective revealed that specificities vary according to intervention goals and the operationalization of adherence versus engagement, with women being more adherent and engaged than men in interventions targeting distress. When focusing on adherence rather than engagement, older patients were more adherent than younger patients.

**Conclusions:**

This review highlights the complexity of adherence to and engagement with digital interventions in cancer care. While some factors, notably comorbidities and low social support, were consistently linked to adherence and engagement, others displayed mixed associations. The review underscores the need for standardizing measures, investigating specific intervention features, and enhancing study quality to optimize digital interventions for patients with cancer. Further research is crucial to better understand and improve adherence to digital health solutions in cancer care.

**Trial Registration:**

PROSPERO CRD42021281028; https://www.crd.york.ac.uk/prospero/display_record.php?RecordID=281028

## Introduction

### Background

Digital interventions include a range of technologies, such as telehealth, mobile health, and web-based platforms that provide health-related information, self-help, support, and monitoring [1]. They have become increasingly popular in recent years as a means of delivering health care services, promoting patient self-care, and improving their health [2], especially for patients with cancer. Indeed, the intricate trajectory of the treatment journey of patients with cancer, spanning diverse health care settings, can be significantly enhanced through the use of digital interventions [3]. By acknowledging the needs encountered by patients with cancer, these interventions proficiently cater to their requirements by providing educational materials, behavior change support, and access to self-help resources [4-6]. Furthermore, digital interventions are pivotal in fostering long-term survivorship care by facilitating the formulation of personalized treatment plans, vigilant monitoring, and advocating for healthy lifestyle choices [7]. More precisely, many digital interventions have been created and tested in the context of cancer care, to improve the patient’s quality of life and symptoms as well as promote healthy behaviors [7-9].

Digital health encompasses a wide range of interventions, from forums to websites, with various objectives (eg, information, sharing of experience, self-assessment, behavior change). Numerous eHealth interventions rely on self-help [10,11] (ie, interventions that can be worked through independently by patients themselves). Patients with cancer hold a positive attitude both toward self-help and eHealth self-help, specifically [12]. Digital interventions with a self-help component seem to be cost-effective in cancer care [13] and research has shown that they could be as efficacious as in-person interventions [14]. This specific type of digital intervention seems to both address patients’ needs and be efficacious in improving their quality of life and overall well-being. However, several studies have reported difficulties in the implementation of digital interventions, several of them being related to patients’ engagement and adherence [15]. Although interrelated, both concepts encompass different experiences and behaviors. Engagement has been defined by Perski et al [16] as both a subjective experience characterized by focused attention, interest, and affect as well as the behaviors associated with this experience. These behaviors include the frequency and duration of use of the digital health intervention. By contrast, adherence to eHealth could be defined as the congruence between the intended use of technology and the effective use by an individual. Moreover, the justification for the intended use should be supported by theory or rationale [17].

Adherence and engagement are often used interchangeably in studies on digital interventions. For example, some operationalize “adherence” to refer to “the more use, the better,” without specifying an intended use. This, of course, brings up measurement issues and disparities [17,18]. Engagement is typically measured with log-ins, time spent on an intervention, or number of clicks [16]. Owing to the confusion between engagement and adherence, the latter is often measured with the same indicators as engagement. However, Sieverink et al [17] noted that previous studies that presented adherence, mostly used a measure of completion of the intervention (eg, number of modules accessed and completed). Indeed, previous work has shown that patients were less likely to be adherent to self-help interventions in comparison with interventions involving real-time interactions [19]. Engagement is an important predictor of the effectiveness of these interventions [14,20-22]. Indeed, the more patients use eHealth, the more it is effective. Although digital interventions have the potential to improve patient outcomes and increase access to health care services, adherence to and engagement with these interventions remain a challenge [23]. It is important to note that the patient’s view of the intervention is essential to its successful implementation [15]. Understanding the factors that influence adherence to and engagement with digital interventions is critical for developing effective interventions.

Few reviews have examined the factors associated with adherence to and engagement with digital interventions. They showed that engagement depended on users’ characteristics, technological aspects, and intervention features [24]. Furthermore, they highlighted that components such as personalized content, push notifications, and quizzes were associated with increased adherence and engagement [25,26]. However, the focus of these systematic reviews limits their reach. Indeed, the 3 systematic reviews that specifically explored adherence to or engagement with digital interventions focused on the intervention features that increased the said adherence and engagement or were conducted in very heterogeneous populations (eg, patients with gynecological problems, caregivers of disabled children) [24-26], without addressing the specificities of cancer care. Another problem lies in the difficulty to define adherence in the context of digital health. In most definitions, use, engagement, and adherence are considered as synonyms [17,27]. While some studies investigate digital health engagement exclusively through log data (eg, number of logins, number of clicks, time spent on a module), other authors argue that adherence should be conceptualized as the degree to which users followed the program as it was designed [27]. In summary, the diversity in the definitions of adherence and engagement makes it challenging to investigate the topic.

In sum, to date, adherence to and engagement with digital health, and particularly to self-help interventions, remains difficult to define, and the factors involved in engagement and adherence have been inadequately investigated, specifically for cancer. Indeed, most systematic reviews published to date on digital health interventions focus on their efficacy [9,28,29] or users’ experience with such interventions [30,31], with a small portion of them mentioning engagement as a secondary objective [19,32].

### This Review

In this systematic review, we aim to identify and synthesize the existing literature on the factors associated with adherence or engagement with digital interventions presenting a self-help component in cancer care. Specifically, we examine the sociodemographic, psychosocial, health-related, and intervention-related factors that influence digital health adherence. We also investigate specificities according to intervention goal, operationalization of adherence and engagement, and intervention type.

## Methods

### Overview

The review was conducted according to the PRISMA (Preferred Reporting Items for Systematic Reviews and Meta-Analyses) guidelines. This systematic review has been registered in the PROSPERO database (CRD42021281028).

### Search Strategy

A comprehensive search strategy was developed by EC and LM to identify relevant studies. The following databases were searched: PubMed, Embase, Cochrane Library, and PsycINFO. The search terms included cancer, eHealth interventions, adherence, and related synonyms. The search strategy is presented in Multimedia Appendix 1. The last systematic review with a similar objective was published in 2011 [24]. Therefore, the search was limited to studies published in English from January 2010 to September 2021.

### Study Selection and Data Extraction

Two reviewers (EC and LM) independently screened titles and abstracts to identify potentially eligible studies through Rayyan software (Rayyan Systems, Inc). Once the blind was off, disagreement was resolved through consensus. Full-text articles were retrieved for studies that met the inclusion criteria. Studies were included if the following criteria were met: (1) they investigated factors associated with adherence to or engagement with digital interventions, (2) said interventions included a self-help component (ie, components that could be worked through independently by patients themselves and implied an active participation from them), and (3) included adult patients with cancer. Studies were excluded if they (1) were not original research (eg, reviews, editorials, and commentaries), (2) did not report empirical data, (3) were qualitative studies, (4) were not intervention trial, (5) only included information modules, (6) included symptom reporting only, without an active component, or (7) included a communication module with health care providers, without an active component.

Three reviewers extracted data from eligible studies using a standardized data extraction form. The following data were extracted: authors, year, country, title, study design, population, intervention type, aim of the intervention, operationalization of adherence and engagement, adherence or engagement measure, intervention duration, study length, primary outcome of interest, analysis, and factors associated with adherence (Multimedia Appendix 2).

### Data Synthesis

A narrative synthesis was conducted owing to the heterogeneity of the included studies in terms of study design, intervention type, and outcome measures. Factors associated with adherence and engagement were synthesized according to their type: sociodemographic factors, psychosocial factors, health-related factors, technology-related factors, and intervention-related factors. We also performed subgroup synthesis according to the intervention goal, operationalization of adherence and engagement, and intervention type.

The quality of the included studies could not be assessed with a standardized evaluation grid because of the variety of designs and objectives. However, we proceeded to a narrative critical appraisal of the methods used across studies. We also quantified the studies for which we identified missing summary statistics.

## Results

### Overview

**Figure 1:**
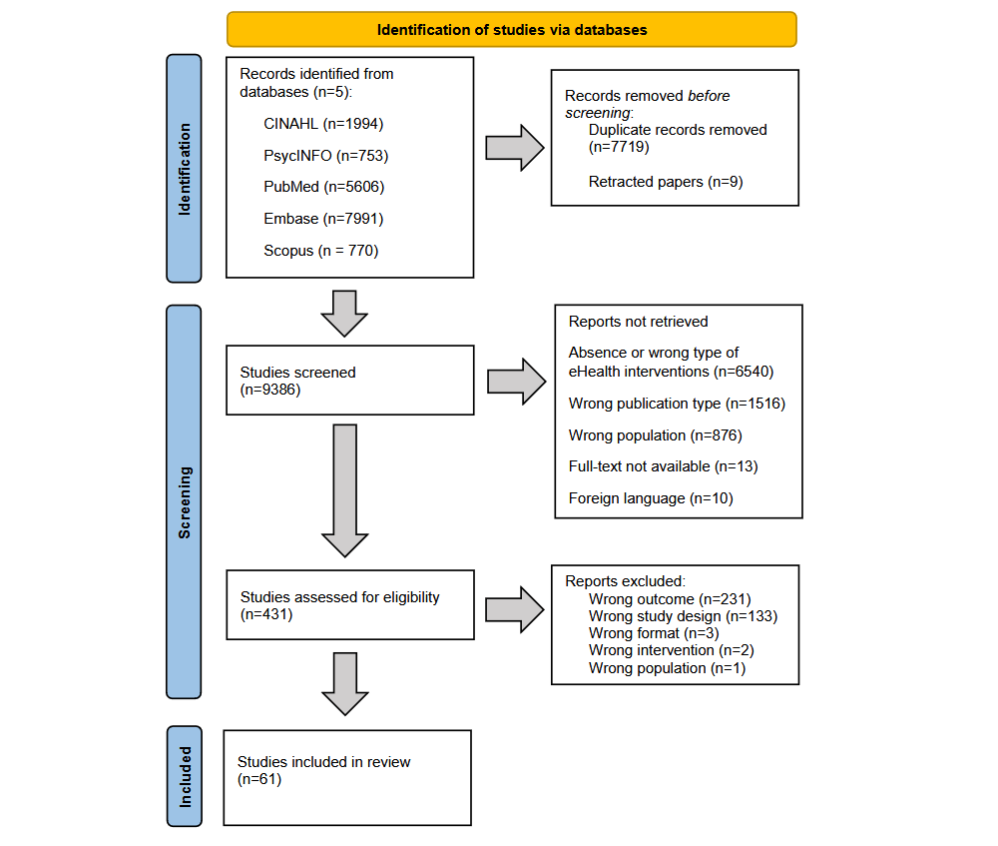
Studies flowchart.

The search identified 9386 potentially eligible studies, of which 61 (0.6%) [33-93] were included in the final review. A detailed flowchart is available (Figure 1).

The interventions included web-based interventions (39/61, 64%), mobile-based interventions (18/61, 30%), other types of digital health interventions, and a combination of different types of technologies (4/61, 7%). The goals of the interventions varied (eg, decreasing distress and behavior change). Regarding the population, breast cancer was the most common cancer type across studies (41/61, 67%). Furthermore, a large portion of studies targeted specifically cancer survivors (25/61, 41%). Adherence and engagement were assessed with several measures in 57% (35/61) of the included studies. The measures used included dropout rates (20/61, 33%), time spent on the intervention (20/61, 33%), number of log-ins (18/61, 30%), number of patients’ actions within the intervention (eg, message sent, clicks; 15/61, 25%), number of pages or modules viewed (12/61, 20%), completion of the intervention or its modules (10/61, 16%), self-reported measures of use (7/61, 12%), number of active days or weeks (6/61, 10%), specific measures linked to the use of wearables (3/61, 5%), and intention to use the application (2/61, 3%). About 10% (6/61) of the included studies used adherence measures specific to their intervention (eg, doing several specific actions, such as creating a user profile and posting >2 messages to a group). Even within these categories, discrepancies in measures are of note (eg, binarization of log-ins measures, completion of the intervention as a whole vs its modules, and number of pages viewed vs view of a specific page). Finally, investigating the determinants of adherence to eHealth was the main objective for 43% (26/61) of the included studies. Factors associated with adherence and engagement included sociodemographic factors, psychosocial factors, health-related factors, and intervention-related factors. Unsurprisingly, adherence was highly associated with other measures of engagement [33-37]. Table 1 summarizes the characteristics of the included studies while Multimedia Appendix 2 presents each study individually in greater detail.

**Table 1:** Descriptive statistics for the included studies (n=61).

		Studies, n (%)
**Type of study**		
	RCT^a^	31 (51)
	Secondary analysis of RCT	15 (25)
	Observational	15 (25)
Studies where most of the patients had breast cancer		41 (67)
Studies on cancer survivors		25 (41)
**Adherence operationalization**		
	Intention	2 (3)
	Engagement	32 (52)
	Adherence without justification	25 (41)
	Adherence with justification	2 (3)
**Measures**		
	Combination	35 (57)
	Dropout rate	20 (33)
	Time spent on the intervention	20 (33)
	Number of log-ins	18 (30)
	Number of actions within the intervention	15 (25)
	Number of page views	12 (20)
	Completion of intervention or modules	10 (16)
	Self-reported measures of use	7 (11)
	Number of active days or weeks	6 (10)
	Measures of wearables	3 (5)
	Intention to use the app	2 (3)
	Specific measures	6 (10)
**Type of intervention**		
	Web-based intervention	39 (64)
	Mobile-based intervention	18 (30)
	Others or combination	4 (7)
**Goal of the intervention**		
	Distress	15 (25)
	Nutrition and physical activity	10 (165)
	Quality of life and symptom management	10 (15)
	Others	14 (23)
	Combination	12 (20)

^a^RCT: randomized controlled trial.

### Sociodemographic Factors Associated With Adherence to and Engagement With Digital Interventions in Patients With Cancer

We found that 38 of 61 (62%) studies investigated the links between sociodemographic factors and engagement with and adherence to digital interventions, with many inconsistent results between studies. First, of the 14 studies concerning age, 6 (43%) showed that older patients tended to be more adherent and engaged than younger ones [34,38-42], 4 (29%) concluded that younger patients were more adherent and engaged [43-46], while 3 (21%) showed no significant associations between age and adherence or engagement [33,47,48]. Finally, 1 (7%) of these studies showed a differential association between age and engagement with digital health interventions; participants’ age was not significantly related to smartwatch-wearing compliance but was significantly and positively correlated with higher symptom rating compliance [49].

Second, of the 13 studies concerning education level, 5 (38%) studies reported that a higher level of education was associated with higher engagement or adherence [38,41,43,50,51], 3 (23%) reported the contrary [34,44,52], and 5 (38%) yielded nonsignificant results [33,39,40,47,53]. One (8%) study reported that patients with some college degree showed the highest decline in engagement compared with high school graduates and college graduates without reporting any statistical tests [39]. Reliable trends regarding economic factors were difficult to determine because of inconsistent results. Among the 5 studies that investigated these links, 2 (40%) showed that being from a privileged background was associated with better adherence and engagement [41,54], while 3 (60%) showed nonsignificant results [33,35,47]. One (20%) study highlighted how income and employment status were associated with the differential use of specific modules [41].

Third, of the 9 studies concerning employment, 4 (44%) studies showed that employed patients were also found to be more adherent and engaged than those who did not work [39,43,50,55], 2 (22%) reported they were less likely to be adherent and engaged [56,57], and 3 (33%) reported nonsignificant results [33,39,53].

Fourth, of the 7 studies concerning gender, 3 (43%) studies showed that women were more adherent or engaged than men [43,50,58], 1 (14%) showed the contrary [34], while 3 (43%) showed no significant association [47-49]. One (14%) study showed that women were more likely to use the printed materials of a hybrid intervention than men [48].

Fifth, of the 5 studies that examined the association between marital status and adherence, 2 (40%) studies suggested that married patients may be more adherent and engaged [38,43], 2 (40%) studies yielded nonsignificant results [39,47], and 1 (20%) showed that married patients were less adherent and engaged [59]. One (20%) study showed that a higher number of people in the household was associated with lower engagement. This same article highlighted that widowed or divorced patients and participants in single households showed a greater decline in engagement, but no statistical test was reported [39].

Sixth, of the 3 studies on race, 2 (67%) studies yielded nonsignificant associations [33,35], and 1 (33%) reported a significant association that showed White women were more likely to use a discussion group module [44].

Finally, 3 studies reported that patients who were more experienced with technology were found to be more adherent and engaged. Although experience with technology was found to be associated with higher use in 2 (67%) studies [33,43], it was not associated with higher use of specific modules [60]. Beyond experience, access to technology was associated with better digital health engagement in 1 (33%) study [35].

### Health-Related Factors Associated With Adherence to and Engagement With Digital Interventions in Patients With Cancer

We found that 30 of 61 (49%) studies reported the links between health-related factors and adherence to digital interventions, with, again, many inconsistent results between studies. First, of the 7 (23%) studies that described the stage of cancer, 3 (43%) studies showed that patients living with a more advanced cancer stage were more adherent and engaged [44,61,62], while 3 (43%) did not show any significant associations [33,41,48]. The last 1 (14%) showed that patients living with cancer stage II had the lowest engagement rate. This same study argued that patients who had breast cancer stage III showed a greater decline in engagement, without reporting any statistical test [39].

Second, of the 5 (8%) studies concerning symptoms, 2 (40%) studies showed that patients with more symptoms tended to be more adherent and engaged [34,63], while 3 (60%) reported no significant associations [39,64,65].

Third, of the 4 (7%) studies concerning comorbidity, patients with comorbidities were found to be less adherent and engaged in 2 (50%) studies [38,60], 1 (25%) study reported nonsignificant associations [41], and in 1 (25%) study, the significance of this association depended on the time of the intervention and the comorbidity measure used [39].

Fourth, of the 3 (5%) studies concerning weight or other related measures, 1 (33%) study showed that patients with a higher percentage of body fat were less adherent and engaged [66], 2 (67%) reported no significant association [39,41], and 1 (33%) study highlighted a greater decline in engagement in participants with high BMI without reporting statistical tests [39].

Fifth, among the 3 (5%) studies that investigated the links between diagnosis date and adherence or engagement, 1 (33%) showed that patients with an older date of diagnosis date had higher engagement [47] and the other 2 (67%) reported no significant results [39,67]. The moment patients are proposed to use the intervention also seems to play a role in their adherence and engagement [35,44,45,52,62]. Only 2 (40%) studies showed no significant associations [33,67]; however, authors used very different indicators (eg, summertime, time since diagnosis, and pre or postsurgery status), which makes it difficult to identify trends.

Sixth, of the 2 studies that examined the association between cancer type, 1 (50%) study showed that patients with breast cancer had higher engagement [62], while 1 (50%) study showed no significant differences in adherence according to cancer type [48]. Finally, 1 (2%) study among the 61 included, although not directly having cancer type as a predictor, demonstrated that the predictors of engagement differed between patients with breast cancer and those with prostate cancer [38].

Seventh, 5 (8%) studies investigated the role of treatments. Four (80%) showed that treatments and medical services could be associated with adherence or engagement [41,42,50,53], but the type of treatments investigated varied greatly (eg, surgery, sleeping medication) and the operationalization of these variables differed across studies (eg, medical service, cycles of chemotherapy). One (20%) study did not show any difference in engagement between patients who underwent chemotherapy and those who did not [39].

Finally, 1 (2%) study showed that patients with cancer had higher engagement scores than participants who did not have cancer [68]. However, in this study, no significant differences emerged in other adherence markers, such as homework completion.

### Psychosocial Factors Associated With Adherence to and Engagement With Digital Interventions in Patients With Cancer

Despite fewer studies examining psychosocial factors (14/61, 23%), they yielded more consistent results. First, of the 13 (93%) studies concerning distress, 6 (46%) studies showed that patients who were distressed were more adherent or engaged [33,44,57,58,69,70], 4 (31%) showed the contrary [35,47,50,71], and 3 (23%) did not report any significant results [36,39,72]. All the studies that showed a positive association used a measure of cancer-related distress [33,44,57,58,69-71]. In comparison, among those that showed the contrary, only 1 (25%) used a cancer-specific measure [47]. Moreover, 1 (8%) of these studies showed how distress was associated with engagement with different modules depending on patients’ gender [71]. One (8%) study showed a differential association depending on the operationalization of engagement (eg, binarization of use vs use of specific modules) [71]. Finally, 1 (8%) study showed that although distress was a predictor of continuous app use, when controlling for gender, this association was no longer significant [58].

Second, of the 7 (50%) studies that investigated the link between quality of life and adherence or engagement, 3 (43%) studies showed that patients with better quality of life were more adherent or more engaged [65,71,73], 1 (14%) reported the contrary [44], and 3 (43%) did not report significant results [39,56,60].

Third, of the 6 (43%) studies investigating social support, 4 (67%) studies showed that greater social support was associated with decreased adherence or engagement [44,60,69,70] and 1 (17%) showed the contrary [34]. Moreover, 1 (17%) of these studies showed a differential association between social constraints and engagement with digital interventions; participants’ social constraints were positively associated with duration of use but negatively with unique module views [33]. As with distress, 1 (17%) study highlighted a different pattern of association between social support and adherence depending on the gender of the participants [60].

Fourth, although self-efficacy was investigated in only 3 (21%) studies, it was found to be positively associated with adherence and engagement in 2 (67%) studies [47,65], while the remaining 1 (33%) reported nonsignificant results [60].

Fifth, positive perceptions of digital interventions (eg, perceived ease of use, perceived usefulness) were consistently associated with better adherence and higher engagement in the 5 (8%) studies that investigated this factor [37,46,59,74,75]. However, 1 (20%) of these studies showed a differential association between intervention perception and adherence; perceived usefulness was positively associated with the intention to use the intervention, but perceived ease of use was not [74].

Sixth, the 3 (5%) studies that investigated the link between information competence and adherence and engagement reported nonsignificant results [33,44,76]. However, 1 (33%) of these 3 studies showed a differential association between information competence and engagement with specific modules; the more patients had high information competence, the less they used “ask the expert” and interactive services, but not information services [44].

Seventh, 3 (5%) studies investigated the links between resistance (such as lack of motivation or difficulty with change) and adherence or engagement. One (33%) of the 3 studies showed a positive association [58], another 1 (33%) showed a negative association [35], and the last 1 (33%) had no significant results [77]. In 1 (33%) study, although resistance to change was a predictor of continuous app use, when controlling for gender, this association was no longer significant [58].

Finally, other psychosocial factors have been found to be associated with adherence or engagement in single studies, such as decisional conflict [76], health perceptions [35], coping (helplessness and anxious preoccupation) [72], personality (openness) [58], previous experience with a similar intervention (mindfulness) [50], fatigue [55], and unmet sexual and physical needs [57]. Other studies investigated different factors without highlighting significant results like therapeutic alliance [78]. One (7%) study stated that patients reporting fatigue showed a greater decline in adherence without reporting statistical tests [39].

### Intervention-Related Factors Associated With Adherence to and Engagement With eHealth Interventions in Patients With Cancer

Twenty-six of 61 (43%) studies investigated the links between intervention-related factors and adherence to digital interventions. First, of the 11 (42%) studies concerning intervention type, most studies compared adherence or engagement with digital intervention with other interventions which made it difficult to summarize these results. Four (36%) studies compared digital interventions with paper pamphlets; 3 (75%) of them showed that participants were more adherent and engaged to the digital version of the intervention [45,79,80], and the last 1 (25%) showed no significant differences between the 2 types of intervention [54]. Two (18%) studies compared digital interventions with usual care; 1 (50%) showed that participants in the digital group engaged more in survivorship care plans than people who did not use the digital intervention [37], while the other 1 (50%) showed no significant differences in dropout rates between the digital group and the usual care group [69]. Two (18%) studies compared interactive digital interventions with information-only portals; 1 (50%) yielded inconsistent results depending on the measure used [81], and the other 1 (50%) had nonsignificant results [72]. Two (18%) studies compared eHealth interventions with face-to-face ones; 1 (50%) showed that participants who participated in the eHealth intervention were less adherent than those who participated in its face-to-face version [78], and the other 1 (50%) did not show any significant differences [34]. Two (18%) studies compared phone interventions with digital ones showing no significant differences [54,66]. Finally, 4 (36%) studies compared 2 different interactive digital interventions; 3 (75%) did not show any significant differences [39,82,83], while 1 (25%) highlighted inconsistent results depending on the chosen measure of engagement [51]. Interestingly, the possibility of interactions between patients yielded nonsignificant associations in the 2 (100%) studies that investigated this topic [74,84].

Second, the link between human support (ie, help from a human with the use of the intervention) and adherence has been studied in only 2 (8%) studies, and the results were conflicting. Of the 2 studies, 1 (50%) showed that human support was associated with increased adherence [85], while the other 1 (50%) showed the contrary [64]. However, it is worth mentioning that 1 (50%) of these studies showed a differential association between human support and engagement and adherence; participants in the technician-guided group logged in more frequently than the self-help group but no significant differences were observed in the proportion of participants who completed lesson 5 between both groups [85].

Third, of the 2 (8%) studies that investigated the effect of time on engagement, 1 (50%) study showed that log-in attrition was significant across the 3 months of the study [33], while the other 1 (50%) showed no significant effect of time on engagement [86].

Finally, although most studies included in this review did not examine the differential use of modules (only 1 study reported no significant differences in module use [87]), a subset of studies investigated the impact of specific features on adherence and engagement. Findings from these studies suggest that a tunneled intervention, in which modules are presented in a fixed sequence, may lead to higher engagement than a free-choice intervention [51]. In addition, reminders were found to be effective in improving adherence and engagement [88,89], regardless of the type of reminder used [90]. Finally, patients were found to consult modules more frequently when informed through interventions tailored to meet their needs [59].

### Intersectional Approach to Adherence to and Engagement With Digital Health Interventions

Given the high heterogeneity of studies and interventions as well as the contrasting results presented above, we examined these data with an intersectional approach. Indeed, we chose to investigate these factors by type of intervention, specifically the types of goals the included studies focused on (eg, physical activity and psychological distress), operationalization of the outcome (ie, engagement and adherence with or without justification for dose), and type of intervention (ie, web-based vs mobile-based). We reported on factors investigated by at least 2 studies in each subcategory.

### Intervention Aims

#### Quality of Life and Symptom Management

A total of 13 (21%) interventions centered on quality of life or symptom management, including 3 (23%) that focused on other aims as well (eg, distress). For interventions that targeted quality of life, 2 (15%) studies examined the association between marital status and adherence and engagement, leading to inconsistent results. One (8%) suggested that married patients had a higher engagement [38], and another 1 (8%) showed contrary results [59]. Regarding comorbidities, patients with comorbidities had a lower engagement in the 2 (15%) studies that investigated this factor [38,60].

Among the 3 studies that investigated the link between quality of life and adherence and engagement, the results were inconsistent. One (33%) of the 3 studies showed that better quality of life was associated with higher adherence [73], another 1 (33%) reported the contrary [44], and the last 1 (33%) did not report significant results [60]. The 2 (67%) studies that investigated social support showed that greater social support was associated with decreased adherence or engagement [44,60].

Regarding intervention-related factors, reminders were found to be effective in improving engagement [88,89]. This was the only factor investigated by >2 studies.

#### Psychological Distress

Twenty-one out of 61 (34%) interventions centered on psychological distress, including 6 (10%) that focused on other aims as well (eg, decisional conflict, self-efficacy). Regarding sociodemographic factors, 1 (33%) study reported that a higher level of education was associated with higher engagement [43], while 2 (67%) others yielded nonsignificant results [33,47]. The 2 (100%) studies that investigated economic background showed nonsignificant results [33,47]. Concerning employment, a reliable trend could not be identified as 1 (33%) study found that employed patients were more adherent than those who did not work [43], another 1 (33%) reported they were less likely to be adherent [57], and the last 1 (33%) reported nonsignificant results [33]. Regarding gender, 2 (67%) studies showed that women were more adherent and engaged than men [43,58], and 1 (33%) showed no significant association [47]. No reliable trend could be identified regarding marital status as 1 (33%) study suggested that married patients may be more adherent [43], another 1 (33%) yielded nonsignificant results [47], and the last 1 (33%) showed that married patients were less adherent [59]. Finally, experience with technology was found to be associated with higher use in 2 (100%) studies [33,43].

Regarding health-related factors, 2 (67%) studies showed that patients living with a more advanced cancer stage were more adherent [61], while 1 (33%) did not show any significant associations [33]. No reliable trend could be identified regarding the time the intervention was proposed to the patients. Indeed, 1 (50%) study showed significant results [62] while the other 1 (50%) showed no significant associations [33].

Concerning distress, 5 (56%) studies showed that patients who were distressed had higher levels of adherence or engagement [33,57,58,69,70], 2 (22%) showed the contrary [47,71], and 2 (22%) did not report any significant results [36,72]. Regarding social support, 2 (100%) studies showed that greater social support was associated with decreased engagement [69,70].

Regarding intervention-related factors, the 2 (100%) studies that compared digital interventions with paper pamphlets showed that participants were more adherent to the digital version of the intervention [79,80]. Two (10%) studies compared interactive digital interventions with information-only portals; 1 (50%) yielded inconsistent results depending on the measure used [81], and the other 1 (50%) yielded nonsignificant results [72].

#### Physical Activity and Nutrition

Twelve out of 61 (57%) interventions centered on physical activity and nutrition, including 2 (17%) that also focused on other aims as well (eg, smoking). First, concerning age, 1 (25%) study showed that older patients tended to engage with the intervention more than younger ones [39], 2 (50%) showed the contrary [45,46], while 1 (25%) study showed no significant associations between age and adherence [48]. No reliable trend could be identified for education; 1 (33%) study reported that a higher level of education was associated with higher engagement [51], 1 (33%) reported the contrary [52], and the last 1 (33%) yielded nonsignificant results [39]. Regarding gender, the 3 (100%) studies that investigated the topic showed no significant association [48,49].

Regarding health-related factors, 1 (50%) study out of 2 did not show any significant associations between cancer stage and engagement [48], while 1 (50%) showed that patients living with cancer stage II had the lowest engagement rate [39]. Concerning symptoms, 1 (50%) study showed that patients with more symptoms tended to have higher engagement while the other 1 (50%) reported no significant associations [39]. Regarding weight or other related measures, 1 (50%) study showed that patients with a higher percentage of body fat had lower engagement [66] and the other 1 (50%) reported no significant association [39]. Finally, the moment patients are proposed to use the intervention also seems to play a role in their engagement among the 3 (100%) studies that investigated this topic [35,45,52].

Regarding psychological factors, no reliable trend regarding distress could be established as it was investigated by only 2 (17%) studies with contradicting results; 1 (50%) showed that more distressed patients had lower engagement [35], and the other 1 (50%) did not report any significant results [39]. However, positive perceptions of digital interventions were associated with better adherence in the 2 (100%) studies that investigated this factor within this subcategory [46,59].

### Operationalization of Adherence and Engagement

#### Overview

We found that 2 out of 61 (3%) studies used measures of intention to continue using the intervention as an outcome. The others were classified according to the classification proposed by Sieverink et al [17]: engagement, adherence with justification for intended use, and adherence without justification for intended use [17]. However, only 2 out of 59 (3%) studies justified the intended use specified in the article. No similarity could be reported as their methods and results differed greatly. Therefore, we only report the results for studies that investigated engagement versus the ones that investigated adherence without justification for the intended use.

#### Engagement

We found that 32 out of 61 (52%) articles used measures of engagement according to the classification proposed by Sieverink et al [17]. First, concerning age, 10 studies investigated this topic: 4 (40%) studies showed that older patients tended to engage more than younger ones [38-41], 3 (30%) studies showed that younger patients engaged more [44-46], while 3 (30%) studies showed no significant associations between age and engagement [33,47,48]. Second, concerning the level of education, 3 (33%) studies reported that higher education was associated with higher engagement [38,41,51], 2 (22%) reported the contrary [44,52], and 4 (44%) yielded nonsignificant results [33,39,40,47]. Among the 4 (13%) studies that investigated economic factors, 1 (25%) showed how being from a privileged background was associated with better engagement [41], while the other 3 (75%) showed nonsignificant results [33,35,47]. Concerning employment, no reliable trend could be identified as the 3 (9%) studies that investigated the topic reached different conclusions; 1 (33%) showed that employed patients were found to have higher engagement than those who did not work [39], 1 (33%) reported they were less likely to engage in the intervention [56], and the last 1 (33%) reported nonsignificant results [33]. The 3 (100%) studies that investigated gender showed no significant association [47,48]. Regarding marital status, 1 (33%) study suggested that married patients may have higher engagement [38], and 2 (67%) studies yielded nonsignificant results [39,47]. For race, 2 (67%) studies yielded nonsignificant associations [33,35], and only 1 (33%) reported a significant association and showed that White women were more likely to use a discussion group module [44]. Finally, experience with technology was found to be associated with higher use in 1 (50%) study [33] but was not associated with higher use of specific modules in another (n=1, 50%) study [60].

Regarding health-related factors, 3 (50%) studies showed that patients living with a more advanced cancer stage had higher engagement [44,61,62], while 3 (50%) did not show any significant associations [33,41,48]. Patients with comorbidities were found to be less engaged in 2 (50%) studies [38,60], 1 (25%) reported nonsignificant associations [41], and in the other 1 (25%), the significance of this association depended on the time of the intervention and the comorbidity measure used [39]. Concerning symptoms, 3 (100%) reported no significant associations [39,65]. One (33%) study showed that patients with a higher percentage of body fat had lower engagement [66], while 2 (67%) reported no significant association [39,41]. No reliable trend could be identified regarding the date of diagnosis as 1 (50%) study showed that patients with an older date of diagnosis had higher engagement [47], and the other 1 (50%) reported no significant results [39]. The moment patients are proposed to use the intervention also seems to play a role in their engagement as shown in 5 studies (83%) [35,44,45,52,62]. Only 1 (17%) study showed no significant associations [33]. Regarding cancer type, 1 (50%) study showed that patients with breast cancer had higher engagement [62], while the other 1 (50%) showed no significant differences in engagement according to cancer type [48]. The association between engagement and treatments or medical services yielded inconsistent results. One (50%) study showed significant differences [41], while another 1 (50%) did not show any difference in engagement between patients who underwent chemotherapy and those who did not [39].

Regarding psychological factors, 4 (40%) studies showed that distressed patients had higher engagement [33,44,69,70], 4 (40%) showed the contrary [35,47,50,71], and 2 (20%) did not report any significant results [39,72]. Two (33%) studies showed that patients with a better quality of life had higher engagement [65,71], 1 (17%) reported the contrary [44], and 3 (50%) did not report significant results [39,56,60]. Concerning social support, 4 (100%) studies showed that greater social support was associated with decreased engagement [44,60,69,70]. Self-efficacy was found to be positively associated with engagement in 2 (67%) studies [47,65], while the remaining 1 (33%) reported nonsignificant results [60]. Positive perceptions of digital interventions were associated with better engagement in the 2 (%) studies that investigated this factor in this subcategory [37,46]. Finally, the 3 (100%) studies that investigated the links between information competence and engagement reported nonsignificant results [33,44,76].

Relative to intervention-related factors, 2 (100%) studies showed that participants had better engagement with a digital version of the intervention compared with paper pamphlets [45,79]. Two (6%) studies compared digital interventions with usual care; 1 (50%) showed that participants in the digital intervention group were more engaged in survivorship care plans than people who did not use the intervention [37], while 1 (50%) study showed no significant differences in dropout rates between the digital group and the usual care group [69]. Two (6%) studies compared interactive digital interventions with information-only portals; 1 (50%) yielded inconsistent results depending on the measure of engagement used [81], and the other 1 (50%) yielded nonsignificant results [72]. Finally, 4 (13%) studies compared 2 different interactive digital interventions; 3 (75%) did not show any significant differences [39,82,83], while the other 1 (25%) highlighted inconsistent results depending on the chosen measure of engagement [51]. Two (6%) studies investigated the effect of time on engagement; 1 (50%) study showed that log-in attrition was significant across the 3 months of the study [33], while the second 1 (50%) showed no significant effect of time on engagement [86]. In addition, reminders were found to be effective in improving engagement [88,89].

#### Adherence Without Justification for Intended Use

We found that 25 out of 61 (41%) articles used measures of adherence without a justification for intended use, according to the classification proposed by Sieverink et al [17].

Regarding sociodemographic factors, 2 (100%) studies showed that older patients tended to be more adherent than younger ones [34,42]. Two (50%) studies showed that employed patients were more adherent than those who did not work [43,55], 1 (25%) reported they were less likely to be adherent [57], and 1 (25%) reported nonsignificant results [53]. Regarding gender, 2 (50%) studies showed that women were more adherent than men [43,58], 1 (25%) showed that men were more adherent [34], while 1 (25%) showed no significant association [49].

Regarding health-related factors, 2 (67%) studies showed that patients with more symptoms tended to be more adherent [34,63], while 1 (33%) reported no significant associations [64]. Two (100%) studies showed that treatments and medical services could be associated with adherence [42,53].

Regarding psychological factors, 2 (50%) studies showed that patients who were distressed were more adherent [58,69], 1 (25%) showed the contrary [71], and 1 (25%) did not report any significant results [36]. Two (100%) studies showed that patients with a better quality of life were more adherent [71,73]. Concerning social support, 1 (50%) study showed that greater social support was associated with decreased adherence [69], and 1 (50%) showed the contrary [34]. One (50%) study showed a positive association between resistance and adherence [58], and the second 1 (50%) showed no significant results [77].

Concerning intervention type, 1 (50%) study showed that participants were more adherent to a digital version of the intervention compared with a paper pamphlet [80], while the other 1 (50%) showed no significant differences between the 2 types of intervention [54]. Two (8%) studies compared eHealth interventions with face-to-face ones; 1 (25%) showed that participants who participated in the eHealth intervention were less adherent than those who participated in its face-to-face version [78], while the other 1 (25%) did not show any significant differences [34]. Second, the results regarding the links between human support and adherence were conflicting. One (50%) study showed that human support was associated with increased adherence [85], and the other 1 (50%) showed the contrary [64].

### Type of Intervention

We chose to investigate factors associated with adherence and engagement according to the type of intervention (web-based or mobile-based). Two interventions could not be classified in either category.

#### Web-Based Intervention

We found that 41 out of 61 (67%) articles reported on web-based interventions, including 2 (5%) that also included other components (ie, CD-ROM, wearables). Regarding sociodemographic variables, 4 (40%) showed that older patients tended to be more adherent and engaged than younger ones [34,39,40,42], 3 (30%) showed the contrary [44-46], while 3 (30%) studies showed no significant associations [33,47,48]. Regarding the level of education, 3 (30%) studies reported that a higher level of education was associated with higher engagement [43,50,51], 2 (20%) reported the contrary [34,44] and 5 (50%) yielded nonsignificant results [33,39,40,47,53]. The studies that investigated economic factors showed nonsignificant results [33,47]. Concerning employment, 4 (50%) studies showed that employed patients were found to be more adherent or had higher engagement than those who did not work [39,43,50,55], 1 (13%) reported the contrary [57], and 3 (38%) reported nonsignificant results [33,39,53]. Two (40%) studies showed that women had higher engagement than men [43,50], 1 (20%) showed the contrary [34], while 2 (40%) showed no significant association [47,48]. One (25%) study suggested that married patients may be more adherent [43], 2 (50%) studies yielded nonsignificant results [39,47], and another 1 (25%) showed the contrary [59]. Regarding race, 1 (50%) study yielded nonsignificant associations [33] and the only 1 (50%) that reported a significant association showed that White women were more likely to use a discussion group module [44]. Although technology experience was found to be associated with higher use in 2 (100%) studies [33,43], it was not associated with higher use of specific modules in another [60].

Regarding health-related factors, 3 (50%) studies showed that patients living with a more advanced cancer stage were more adherent [44,61,62], while 2 (33%) did not show any significant associations [33,48]. The last 1 (17%) showed that patients living with cancer stage II had the lowest adherence rate [39]. Patients with comorbidities were found to be less adherent in 1 (50%) study [60], and in another 1 (50%), the significance of this association depended on the time of the intervention and the comorbidity measure used [39]. Concerning symptoms, 2 (50%) studies showed that patients with more symptoms tended to be more adherent or more engaged in the intervention [34,63], while 2 (50%) reported no significant associations [39,64]. Concerning weight or other related measures, 1 (50%) study showed that patients with a higher percentage of body fat had lower engagement [66], and 1 (50%) reported no significant association [39]. Among the 3 (7%) studies that investigated the link between diagnosis date and adherence and engagement, 1 (33%) showed that patients with an older date of diagnosis date had higher engagement [47], and the other 2 (67%) reported no significant results [39,67]. The moment patients are proposed to use the intervention also seems to play a role in their engagement (3/5, 60%) [44,45,62]. Only 2 (40%) studies showed no significant associations [33,67]. Two (5%) study examined the association between cancer type, 1 (50%) study showed that patients with breast cancer had higher engagement [62], while 1 (50%) study showed no significant differences in engagement according to cancer type [48]. Three (75%) studies showed that treatments and medical services could be associated with engagement [42,50,53], but 1 (25%) study did not show any difference in engagement between patients who underwent chemotherapy and those who did not [39].

Regarding psychological factors, 5 (50%) studies showed that patients who were distressed had higher engagement or adherence [33,44,57,69,70], 2 (20%) showed the contrary [47,50], and 3 (30%) did not report any significant results [36,39,72]. Of the 3 (7%) studies that investigated the links between quality of life and adherence, 1 (33%) reported that patients with a lower quality of life had lower engagement [44], and 2 (67%) did not report significant results [39,60]. Concerning social support, 4 (80%) studies showed that greater social support was associated with decreased adherence or engagement [44,60,69,70], and 1 (20%) showed the contrary [34]. Self-efficacy was found to be positively associated with engagement in 1 (50%) study [47], while the remaining 1 (50%) reported nonsignificant results [60]. Moreover, the 3 (100%) studies that investigated the links between information competence and adherence or engagement reported nonsignificant results [33,44,76]. Regarding resistance, 1 (33%) study showed a positive association with adherence [58], another 1 (33%) showed a negative one [35], and the last 1 (33%) showed no significant results [77].

Concerning intervention-related factors, the 2 (100%) studies that compared web-based interventions with paper pamphlets showed that participants had higher engagement or adherence with the digital version of the intervention [45,80]. Two (5%) studies compared interactive digital interventions with information-only portals. One (50%) study yielded inconsistent results depending on the measure used [81], and the other 1 (50%) yielded nonsignificant results [72]. Two (5%) studies compared eHealth interventions with face-to-face ones; 1 (50%) showed that participants who participated in the eHealth intervention were less adherent than those who participated in its face-to-face version [78], while the other 1 (50%) did not show any significant differences [34]. Finally, 2 (5%) studies compared 2 different interactive digital interventions; 1 (50%) did not show any significant differences [39], while the other 1 (50%) highlighted inconsistent results depending on the chosen measure [51]. Regarding human support the results were conflicting. One (50%) study showed that human support was associated with increased adherence [85], and the other 1 (50%) showed the contrary [64]. Finally, reminders were found to be effective in improving engagement [88,89].

#### Mobile-Based Intervention

We found that 18 out of 61 (30%) articles reported on mobile-based interventions. Regarding sociodemographic factors, 2 (67%) studies showed that older patients tended to engage less with the interventions than younger ones [38,41], and 1 (33%) showed the contrary [46]. Two (50%) studies reported that higher education was associated with higher engagement [38,41], 1 (25%) reported the contrary [52], and 1 (25%) yielded nonsignificant results [39]. Moreover, 2 (100%) studies showed how being from a privileged background was associated with better adherence or engagement [41,54]. Reliable trends could not be established concerning employment as 1 (33%) study showed that employed patients were found to be more engaged than those who did not work [39], 1 (33%) reported the contrary [56], and 1 (33%) reported nonsignificant results [39]. One (50%) study showed that women were more adherent than men [58], and the other 1 (50%) showed no significant association [49]. One (50%) study suggested that married patients had higher engagement [38], while the other 1 (50%) yielded nonsignificant results [39].

Among health-related factors, patients with comorbidities had a lower engagement in 1 (50%) study [38] and 1 (50%) reported nonsignificant associations [41]. The moment patients are proposed to use the intervention also seems to play a role in their adherence [35,52].

One (33%) showed that distressed patients were more adherent [58], while 2 (67%) showed the contrary [35,71]. Two (67%) studies showed that patients with better quality of life had higher engagement [65,71], and 1 (33%) did not report significant results [56]. Positive perceptions of digital interventions were consistently associated with better adherence and engagement in the 4 (100%) studies that investigated this factor [37,46,74,75]. One (50%) study showed a positive association between resistance and adherence [58], while another 1 (50%) showed a negative association [35]. Regarding intervention-related factors, 1 (25%) study showed that participants were more adherent to the digital version of the intervention than to a paper pamphlet [79], and the other 1 (25%) showed no significant differences between the 2 types of intervention [54]. Finally, 2 (50%) studies did not show any significant differences [82,83].

### Quality of the Included Studies

The quality of the included studies could not be assessed with a standardized evaluation grid, because for several studies, examining the factors associated with adherence was a secondary objective. Therefore, the quality of the method used to assess this objective may not reflect the overall quality of the articles examined.

The results reported in the previous sections varied greatly, including in the same study, depending on the operationalization of the factors under study (eg, social support vs social constraints), their measures (eg, dichotomization of a variable), the way adherence was conceptualized (eg, active app days, percent of messages read) [37], as well as the composition of the sample under investigation (eg, samples composed of people aged ≥65 years [40]). Moreover, some of these results may be imputable to other confounding variables (eg, cancer type and gender).

Finally, it is noteworthy that 15 of the 61 (25%) articles reported a nonsignificant association between some variables under study and adherence, without specifying which ones. This may lead to an overestimation of the effects of said variables, especially sociodemographic and health-related ones [27,35,36,54,56,60,61,69,70,72,77,78,91-93].

## Discussion

### Principal Findings

This review aimed to highlight factors associated with adherence to digital interventions in cancer care. Despite the large number of articles included in this review, most results were heterogeneous across studies. For illustration, sociodemographic factors were the most investigated, but our review showed that these had the most inconsistent results. This may be due to the heterogeneity in the types of intervention under study (ie, duration, objective, and format), the diversity of the populations included (eg, adolescents and young adults, patients with breast cancer), as well as the inconsistency in the measures of adherence (eg, log-ins, time spent, self-reported measures), and its predictors. However, it is also important to note that some results were consistent across most studies. For example, the most adherent patients to digital health were those who were older, without comorbidities, with a positive perception of the intervention, and a low level of social support. Given the large number of factors covered by this review and the heterogeneity of the results, this discussion will focus exclusively on the most surprising results as well as those that were most widely agreed upon in the included studies.

Regarding health-related factors, the factor on which there is the greatest consensus is the presence of comorbidity. Indeed, the association between comorbidities and low adherence to digital health is noteworthy. Similar results have been found in previous studies in older adults and patients living with rheumatoid arthritis [94,95]. The authors highlight that some comorbidities may prevent access to digital interventions (eg, cognitive functioning and vision problems) [95]. Another explanation for these results may be that current digital interventions in cancer care do not address the complexity of these patients’ health and care journeys [96]. These data suggest that the presence or absence of comorbidities must absolutely be taken into account in the conception of adequate digital interventions for patients with cancer.

Regarding psychosocial factors, the results concerning the effect of distress level on adherence were surprising. While distress was found to be positively associated with adherence to digital interventions in little more than half the studies, the results remained inconsistent across other studies. These contradictory results could be explained by the variability of definitions and measures of distress. In patients presenting a mental illness, adherence to digital interventions for depression and anxiety is often low to moderate and predicted by their severity [97-100]. Symptoms of such disorders (eg, anhedonia, lack of motivation) could hinder adherence to these interventions. In the context of cancer, we highlighted that all the studies that showed that higher distress was associated with increased adherence used cancer-specific measures of the concept. Here, the association with distress may be more of an indicator of the need for support regarding their cancer experience than severe symptoms of psychological distress, or even mental illness. In other words, cancer-related distress is an indicator of cancer-related needs; the more patients report such needs, the more likely they are to use digital interventions. The association between distress and adherence to digital health highly depends on how distress is conceptualized (eg, depression, cancer-related distress, anxiety). Consequently, identifying the type and level of distress of patients is crucial to addressing their needs and improving their adherence to the digital interventions offered.

Still among the psychosocial factors, one predictor of adherence that seemed to enjoy consensus in the studies included in this review was social support. More specifically, most studies have shown that low social support was associated with better adherence. This is congruent with studies that showed that cancer patients with low social support were more likely to seek health information on the web [101]. Connection with peers is part of the experience of digital health users, and some interventions include components to foster interactions between them [31]. Indeed, they can successfully improve perceived social support in patients with cancer [102]. Meeting similar people through a shared digital intervention could create a sense of community and decrease feelings of isolation [103,104]. However, not everyone is comfortable with social network features, so these aspects may be less relevant for people who already have a satisfying level of social support [104]. Finally, social support has been linked to better psychosocial outcomes and self-management behaviors in patients [105-107]. Consequently, patients with high levels of social support may be less inclined to use such interventions.

The adoption of an intersectional lens allowed us to highlight specificities according to intervention goals and operationalization of adherence versus engagement. For interventions targeting distress in patients with cancer, gender might play a more important role than in other types of intervention, with women being more adherent and engaged than men. This might be explained by more negative attitudes toward mental health interventions in men [108,109]. Similarly, patients who are distressed seem to be more adherent to distress-focused interventions than other types of interventions. This may be explained by the relevance of such interventions to their needs. However, these results remain inconsistent across studies. Finally, when focusing on adherence rather than engagement, older patients were more adherent than younger patients. This aligns with previous research highlighting that older patients are more adherent than their younger counterparts [110].

### Limitations

This systematic review has limitations worth mentioning. The main one is the significant heterogeneity of the articles included in this review, both conceptually and methodologically. From a conceptual point of view, the absence of a consensus on the definition of adherence in eHealth is a major obstacle. This echoes previous research highlighting a lack of consistency in the definition of this concept in the context of digital health [17]. Due to the wide variation of terms used to refer to adherence (eg, engagement, use, and usage), some relevant studies may not have been included. From a methodological point of view, the analysis of the included studies relies on the content of the articles, yet some of them presented incomplete data (eg, the details of the results and the nonsignificant associations with adherence were not reported). This may lead to an overestimation of the effect of some factors. Examining factors associated with adherence was the main objective of less than half of these studies which may explain the lack of details in some articles. In the future, it seems essential to better conceptualize adherence and to deepen the research into its determinants. Finally, our review included studies with patients with all types of cancers and at different stages of cancer. However, breast cancer was overrepresented in our review which may affect the external validity of our results. In future research, it could be interesting to target a particular type of cancer.

Despite these limitations, we may provide recommendations for the development of future digital interventions targeting patients with cancer. Personalization, specifically, seems to be essential. The platform should consider individual needs, including age, comorbidities, distress type (ie, cancer-specific or nonspecific), and levels. It should target isolated patients to provide tailored support and address gender-specific preferences (eg, regarding mental health). Usability and accessibility are essential, with continuous evaluation for ongoing improvement. Such an intervention would enhance patients’ adherence and engagement, and ultimately, patient outcomes.

### Conclusions

In summary, this systematic review examined factors associated with digital health adherence, aiming to provide a comprehensive understanding of the current state of research in this field. Our analysis revealed several key findings that shed light on the complexity of eHealth adherence. The results underscore the importance of health-related factors and psychosocial factors in predicting adherence. More specifically, the presence of comorbidities and the level of social support appear to be important factors to consider in ensuring patient adherence to digital interventions. However, our review also highlights the need for further investigation in this area, particularly by studying the effects of promising but poorly considered factors, such as self-efficacy. Finally, to gain a clear understanding of the factors involved in adherence to digital interventions for patients with cancer, it seems essential that future research should pay more attention to investigating the effects of specific features (eg, gamification, peer-support modules), standardizing other factors (eg, human support, comparison to other interventions), and homogenization of adherence measurements to enhance study quality. For example, a significant number of studies did not report which variables they investigated when they did not yield significant results. By addressing these gaps and limitations, future research can contribute to improving digital interventions and ultimately enhancing patient outcomes in this digital health care era.

## Appendix

Multimedia Appendix 1 Search strategies.

Multimedia Appendix 2 Descriptive table of the included studies.

Multimedia Appendix 3 PRISMA 2020 Checklist.

## Abbreviations

PRISMA: Preferred Reporting Items for Systematic Reviews and Meta-Analyses
