# Correction: Factors Associated With Digital Intervention Engagement and Adherence in Patients With Cancer: Systematic Review

**DOI:** 10.2196/71370

**Published:** 2025-01-23

**Authors:** Lucile Montalescot, Louise Baussard, Elodie Charbonnier

**Affiliations:** 1 APSY-V Université de Nîmes Nîmes France; 2 Laboratoire de Psychopathologie et Processus de Santé Université Paris-Cité Boulogne-Billancourt France

In “Factors Associated With Digital Intervention Engagement and Adherence in Patients With Cancer: Systematic Review” (J Med Internet Res 2024;26:e52542) the authors noted one error.

The following affiliation has been added for all authors:

APSY-v, Université de Nîmes, Nîmes, France

Meanwhile, the following affiliation has been removed for authors LB and EC:

Laboratoire de Psychopathologie et Processus de Santé, Université Paris-Cité, Boulogne-Billancourt, France

Additionally, the Acknowledgments section has been updated to reflect LM's institutional affiliations, from:

This work was funded by myCharlotte.

To:

This work was funded by myCharlotte. LM was affiliated with the Université de Nîmes at the time of the review, and is currently affiliated with the Université Paris-Cité.

The correction will appear in the online version of the paper on the JMIR Publications website on January, 23, 2025 together with the publication of this correction notice. Because this was made after submission to PubMed, PubMed Central, and other full-text repositories, the corrected article has also been resubmitted to those repositories.

