# Supplementary material for: Factors Associated With Digital Intervention Engagement and Adherence in Patients With Cancer: Systematic Review
Source: J Med Internet Res. 2024 Dec 11;26:e52542. doi: 10.2196/52542 (PMC11669875; doi:10.2196/52542)
Supplement: Multimedia Appendix 1 [file jmir_v26i1e52542_app1.docx]

**Textbox S1**: Search strategy

**PubMed**: (adheren*[tiab] OR complian*[tiab] OR feasab*[tiab] OR accept*[tiab] OR use[tiab] OR user*[tiab] OR usage[tiab] OR adopt*[tiab] OR implement*[tiab] OR intent*[tiab] OR engage*[tiab]) AND (ehealth[ti] OR mhealth[ti] OR telehealth[ti] OR telemedicine[ti] OR application [ti] OR app[ti] OR smartphone[ti] OR mobile[ti] OR web[ti] OR internet[ti] OR digital health[ti] OR telemedicine[ti]) AND (onco*[tiab] OR cancer*[tiab]) ~~(adheren*[tiab] OR complian*[tiab] OR feasab*[tiab] OR accept*[tiab] OR use[tiab] OR user*[tiab] OR usage[tiab] OR adopt*[tiab] OR implement*[tiab] OR intent*[tiab]) AND (ehealth[ti] OR mhealth[ti] OR telehealth[ti] OR telemedicine[ti] OR application [ti] OR app[ti] OR smartphone[ti] OR mobile[ti] OR web[ti] OR internet[ti] OR digital health[ti] OR telemedicine[ti]) AND (onco*[tiab] OR cancer*[tiab])~~

**Embase**: ((adheren* OR complian* OR feasab* OR accept* OR use OR user* OR usage OR adopt* OR implement* OR intent* OR engage*) AND (ehealth OR mhealth OR telehealth OR telemedicine OR application OR app OR smartphone OR mobile OR web OR internet OR digital health OR telemedicine) AND (onco* OR cancer*))

**Cochrane Library**: (adheren* OR complian* OR feasab* OR accept* OR use OR user* OR usage OR adopt* OR implement* OR intent* OR engage*) AND (ehealth OR mhealth OR telehealth OR telemedicine OR application OR app OR smartphone OR mobile OR web OR internet OR digital health OR telemedicine) AND (onco* OR cancer*)

**PsycINFO**: ((adheren* OR complian* OR feasab* OR accept* OR use OR user* OR usage OR adopt* OR implement* OR intent* OR engage*) AND (ehealth OR mhealth OR telehealth OR telemedicine OR application OR app OR smartphone OR mobile OR web OR internet OR digital health OR telemedicine) AND (onco* OR cancer*))
